# Dissonance in Young Adult Cigarillo Users’ Categorization of Concept Flavored and Unflavored Products

**DOI:** 10.3390/ijerph19127219

**Published:** 2022-06-13

**Authors:** Catherine C. Osborn, Jessica P. Suratkal, Stephanie N. Pike Moore, Sarah Koopman Gonzalez, Kymberle L. Sterling, Amanda J. Quisenberry, Elizabeth G. Klein, Erika S. Trapl

**Affiliations:** 1 Department of Population and Quantitative Health Sciences, School of Medicine, Case Western Reserve University, Cleveland, OH 44106, USA; catherine.osborn@case.edu (C.C.O.); jessica.suratkal@case.edu (J.P.S.); stephanie.pike@case.edu (S.N.P.M.); sarah.koopman@case.edu (S.K.G.); 2 Department of Health Promotion and Behavioral Sciences, University of Texas Health Sciences Center, School of Public Health, Dallas, TX 77054, USA; kymberle.l.sterling@uth.tmc.edu; 3 Department of Health Behavior, Roswell Park Comprehensive Cancer Center, Buffalo, NY 14263, USA; amanda.quisenberry@roswellpark.org; 4 Division of Health Behavior and Health Promotion, College of Public Health, The Ohio State University, Columbus, OH 43210, USA; klein.232@osu.edu

**Keywords:** cigarillos, young adults, regulatory science, flavors

## Abstract

This study asks young adult cigarillo users to categorize their preferred flavor in order to examine user consensus and potential methodological and regulatory implications of flavor name-based categorization systems. Young adult (21–28 years) cigarillo users (*n* = 426) named and categorized their favorite cigarillo flavor into one of seven categories: Fruit, Sweet and Candy, Mint, Alcohol, Menthol, Tobacco, and Other. Flavor responses were coded as characterizing (ex: Grape, Wine) or concept (ex: Jazz, Diamond) flavors. Variation within and between categories was assessed, including the presence of concept flavors and the placement of flavors in multiple categories. Of the 66 unique flavor names provided, participants placed 20 (30.1%) in more than one flavor category. Most of the Tobacco (76.9%) and Other (69.2%) flavor names appeared in multiple categories. The majority of flavor names in the Tobacco (69.2%) and Other (61.5%) categories were concept flavors. Concept flavors were placed in multiple categories (45.0%) twice as often as characterizing flavors (23.9%). This study has identified dissonance among cigarillo users’ flavor categorizations, particularly for concept flavored and unflavored products. Flavor names may obscure how and whether a product is flavored. Research on and regulation of flavored tobacco products should classify products by flavor additives rather than by name alone.

## 1. Introduction

Tobacco use is a leading cause of mortality worldwide, associated with more than 8 million deaths each year [1]. Flavor additives in tobacco products contribute to this issue by influencing tobacco use in youth and young adults [2]. Young adults perceive flavored tobacco products as “less risky” than unflavored products [3], although flavored products often have more harmful constituents, such as volatile organic hydrocarbons, increased tobacco mass, and total particulate matter [4]. Additionally, flavors added to tobacco products can help ameliorate the unfavorable tastes of tobacco [5] and create a positive experience associated with flavor [6].

Cigarillos are the most commonly sold cigar product in the United States, typically containing three grams of tobacco; they have no filter, are 3–4 inches long, and are slimmer than traditional large cigars [7]. Cigarillos are predominantly used by the most vulnerable population groups, including youth and young adults, those who identify as Black or Hispanic or female, and those with lower socioeconomic status [8,9,10,11]. Product initiation with flavored cigarillos is particularly common, with 65% of youth and young adult cigarillo users reporting the first cigar product they used was flavored [2]. Existing research regarding flavored tobacco preferences and categories focuses heavily on electronic nicotine delivery systems (ENDSs) [12,13,14,15], with a significant gap in research related to cigarillos despite similar rates of use for both products among young adults [2]. Additional research into the factors contributing to cigarillo initiation and smoking experiences is necessary to inform effective policies aimed at reducing tobacco use and mediating disparities in usage among vulnerable populations.

Although the majority of cigarillos sold in the United States are flavored [16], some cigarillo users are unsure of the flavor of their product. The Population Assessment of Tobacco and Health (PATH) Study found that 16.5% of current cigarillo users were unsure whether they used flavored products [2]. When asked to characterize the flavor they usually used, the majority of young adult cigarillo users responded they were unsure [17]. Not knowing how to categorize their typically used cigarillo flavors may align with the emergence of non-characterizing flavors in the tobacco market, such as concept flavors.

Researchers define concept flavors as those whose names do not characterize the way the product tastes [16,18,19]. For example, “Strawberry” is a characterizing flavor because a strawberry-flavored cigarillo tastes like the fruit. Conversely, the cigarillo flavor name “Jazz” does not readily depict a food or beverage product. Rather, jazz is a music genre that originated in New Orleans. The emergence of concept flavors presents challenges for researchers in categorizing and studying flavored tobacco products. Researchers in previous studies have organized cigarillo flavors—concept and characterizing—into categories based on flavor names as well as product descriptions and user reviews [20,21,22]. Researchers do not always agree on flavor taxonomies or how to sort flavors. For example, different researchers have defined “Jazz” as unflavored [20] as well as a fruit flavor [21]. Other research has relied on cigarillo users self-categorizing their flavor product use without collecting flavor name information [16]. Differences in how concept flavors are categorized in research—either as a standalone group or sorted into characterizing categories—can lead to confusion when comparing study outcomes. 

Classification of tobacco flavors has implications on tobacco regulatory policy. Of those countries that have implemented tobacco flavor restrictions, many have focused specifically on characterizing flavors [23]. Similarly, in the U.S., a policy proposed in 2022 seeks to implement restrictions on characterizing flavors in cigar products [24]. Across these policies, there is no consistent definition as to what exact constituents or additives and their subsequent thresholds create a distinctive characterizing flavor as opposed to a concept or non-flavored/unflavored product, which may dilute the intended public health impact of these policies.

Given cigarillo users’ uncertainty about their own flavor use [2] and inconsistencies in how researchers sort and categorize cigarillo flavors [20,21,22], the purpose of this study is to explore cigarillo users’ own perception and categorization of flavors, particularly across characterizing and concept flavor categories. 

## 2. Materials and Methods

### 2.1. Participant Recruitment

**Figure 1:**
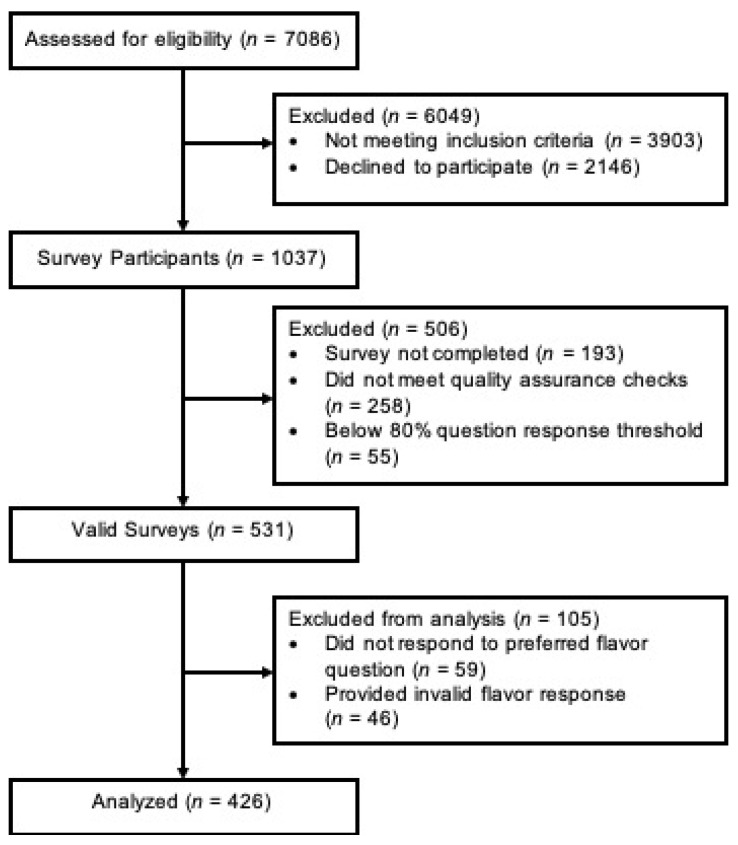
Sample recruitment and analysis.

We recruited young adult cigarillo users for an online survey about cigarillo use and flavor preferences. Participants were recruited between October 2020 and April 2021, first from the Young Cigarillo User (YCU) study sample [25,26] and then through targeted advertisements on the social media platforms Facebook and Instagram. We oversampled sexual and gender minority women, a marginalized population known to have higher cigarillo use [27], to allow for comparisons to cisgender heterosexual women. Interested individuals completed an online screener (*n* = 7086) (Figure 1). Eligible participants had to have lived in the United States, be 21–28 years of age, and report the use of at least two cigarillos in the past 7 days. 

Participants meeting the screening criteria (*n* = 3183) received an email link to the survey administered through Qualtrics, an online survey platform [28]. Of those invited, 1037 participants began the survey, and 844 completed it. Quality control measures included removal of responses with duplicate IP addresses, surveys determined to be outside of the U.S., or respondents who missed more than two of the three attention check questions [29,30]. Participants who completed the survey and correctly answered the attention check questions received a USD 15 e-gift card. Overall, 531 of the survey responses met quality assurance checks and were deemed valid, with respondents answering 80% of the questions per guidelines from the American Association for Public Opinion Research (AAPOR) [31]. This protocol was reviewed and approved by the Institutional Review Board of Case Western Reserve University.

### 2.2. Measures

Participants responded to the open-ended item, “What is your favorite flavor for smoking a cigarillo?” They next responded to the multiple-choice question “In which category does your top flavor choice fit?”, with the options “Fruit (such as cherry, strawberry, apple)”, “Sweet and Candy (such as honey, vanilla, chocolate)”, “Mint (such as mint, peppermint, spearmint)”, “Alcohol (such as bourbon, piña colada, wine)”, “Menthol”, “Tobacco”, and “Other”. Options were developed based on the work of Nyman et al. (2018) [3], with some categories combined to reduce the number of choices based on qualitative feedback during survey development. Mint and menthol were presented as separate options to determine if they were similarly endorsed by participants.

### 2.3. Data Organization

#### 2.3.1. Determining Response Validity

Of 531 survey participants, 472 (88.9%) answered the favorite flavor question. Two researchers independently coded these responses as valid or invalid. The coders considered responses invalid when the participant gave more than one flavor as a response (e.g., “Peach and Chocolate”), provided only the brand rather than a flavor (e.g., “Swisher”, “Backwoods”), named a category of flavors (e.g., “Fruit flavors and dessert flavors are my favorites”), or made a comment not specific to a flavor name (e.g., “The scent”). Consistent with previous research, when it was not clear if the response was a flavor name (such as “Straight Up”), the coders searched manufacturer and retailer websites to determine response validity [32]. After coding validity independently, the coders reconciled disagreements to determine a final valid list of favorite flavor responses. Agreement before reconciliation was high (kappa = 0.952), with only four cases of disagreement. After reconciliation, coders deemed 426 responses valid for a response rate of 80.2% among sample participants.

#### 2.3.2. Coding Characterizing vs. Concept Flavors

Two researchers independently coded the valid flavor responses as “characterizing” or “concept” flavors. Flavors named after non-tobacco items such as fruit, candy, or alcohol (“Mango”, “Chocolate”, “Wine”) or unflavored tobacco (“Tobacco”, “No Flavor”, “Regular”) were coded as characterizing. Flavors with non-explicit names such “Jazz”, “Jamaican Blaze”, or “Silver” were coded as concept flavors [15]. We followed Gammon et al. (2019), coding flavors with the primary descriptor “Sweet” as concept flavors [21]. Before reconciling disagreements in the coding of concept flavors, the coders had a high level of agreement (kappa = 0.887). The 12 cases of disagreement were combination flavor names, including both characterizing and non-characterizing descriptors, such as “Mint Fusion”, “Berry Fusion”, or “Banana Smash.” Following Rogers et al. (2019), these combination names were coded as characterizing [33].

#### 2.3.3. Grouping Responses into Distinct Flavors

Within each of the seven flavor categories, we counted the number of valid responses and the number of distinct flavors. Responses were grouped by flavor name, regardless of whether the brand name was provided by the participant. For example, “Jazz black and mild” and “Jazz” were both classified as “Jazz”.

We considered variations in flavor names as distinct flavors because prior research had identified variations in the chemical composition of cigarillos with similar flavor names [17,19]. For example, the following pairs are considered different flavors: “Red Berry” and “Berry Fusion”, “White Grape” and “Grape”, “Red Sweet” and “Green Sweet”, and “Russian Cream” and “Irish Cream”. We collapsed flavor names indicating a preference for unflavored tobacco into a single flavor. The responses for this flavor included “Original”, “No flavor”, “Plain”, “Unflavored”, “Regular”, and “Tobacco” as these descriptors characterize the tobacco itself [16]. 

The final list of distinct flavors is shown in Table S1.

### 2.4. Data Analysis

#### 2.4.1. Variation within Favorite Flavor Categories

Once the number of distinct flavors in each category was determined, we assessed the variation within categories. We calculated the variation by dividing the number of distinct flavors by the number of responses using the following equation:

Flavor variation = (# Flavors in Category)/(# Responses in Category)

Categories with lower variation ratios indicate higher levels of agreement in favorite flavor and category placement. Categories with higher variation ratios indicate that fewer participants within the category share the same favorite flavor. We assessed the list of distinct flavors within each category for the presence of concept flavors and characterizing flavors with names that appeared to the research team to fit in different categories.

#### 2.4.2. Variation in Concept Flavors within and across Categories

For each category and overall, we calculated the percent of concept flavor responses and distinct concept flavors. We assessed the list of distinct concept flavors to identify possible subcategories.

#### 2.4.3. Variation in Placement of Favorite Flavors in Flavor Categories

To understand whether there was consensus on how participants categorize flavor names, we identified flavors appearing in multiple categories. The percent of flavors appearing in multiple categories out of the total number of distinct flavors in a category was calculated. Categories with higher percentages indicate that more of the flavors in that category also appear in other flavor categories. To determine if there were differences in how participants agreed on the categorization of concept flavors versus characterizing flavors, we calculated and compared the percent of concept flavors appearing in multiple categories to the percent of characterizing/tobacco flavors appearing in multiple categories.

## 3. Results

### 3.1. Sample Demographics and Multiple Tobacco Product Use

The demographic composition of the sample of participants who provided a flavor name (*n* = 426) is described in Table 1. Overall, 61.3% of the sample were non-white, 78.9% were non-Hispanic, and 56.1% were women, with 61.4% of female participants identifying as a sexual and/or gender minority. About three-quarters of participants reported the ever use of non-menthol cigarettes (72.9%), menthol cigarettes (80.5%), e-cigarettes (69.7%), and hookahs (73.0%), and over 40% of participants reported the current use of non-menthol cigarettes (41.4%), menthol cigarettes (47.6%), and e-cigarettes (46.3%).

### 3.2. Favorite Cigarillo Flavors and Flavor Categories

The participants provided 66 unique favorite flavors. The most frequently preferred flavors were “Grape” (8.9%), “Wine” (8.4%), and “Tobacco/Regular/Plain” (8.2%). Of the 66 favorite flavors, 27 (40.9%) were preferred by only one participant, including “Coffee”, “Orange”, and “Red Sweet” (Table S1). Participants most frequently placed their favorite cigarillo flavor in the Fruit (41.5%) or Sweet and Candy (18.3%) categories, with Menthol (5.9%) and Other (4.2%) being the least frequently preferred categories (Table 2). Flavor variation, the ratio of flavors in a category over the number of responses in the category, was lowest in the Mint (0.14) and Fruit (0.17) categories, indicating higher agreement in flavor preference and placement. Meanwhile, there was high flavor variation within the Other category (0.72), with 18 responses grouped into 13 distinct flavors.

#### 3.2.1. Variation within the Fruit Category

Among participants who categorized their favorite cigarillo flavor under the Fruit category, “Grape” was the most frequently selected flavor response (20.3%), followed by “Strawberry” (16.4%) and “Mango” (9.6%). Within the 30 distinct flavor names in the Fruit category, 21 flavors (70.0%), representing 93.2% of the Fruit-categorized responses, included a fruit-characterizing descriptor. The remaining nine non-fruit characterized flavors were coded as either concept flavors (e.g., “Tropical Fusion”, “Jazz”, or “Diamond”) or those appearing to belong in other categories based on their characterizing descriptors (e.g., “Irish Cream” or “Wine”).

#### 3.2.2. Variation within the Sweet and Candy Category

Of the 22 distinct flavors placed in the Sweet and Candy category, the most frequently selected favorite cigarillo flavor was “Vanilla” (25.6%), followed by “Honey” (21.8%), “Sweet” (10.3%), and “Jazz” (10.3%). Participants categorized flavor names that characterized dessert foods, such as “Candy” or “Chocolate”, as well as flavor names with the descriptor “Sweet”, including “Sweet”, “Black Sweet”, and “Green Sweet”, into the Sweet and Candy category. We also identified spice-related names such as “Vanilla” and “Clove” in this category. Although they contained a fruit descriptor, a single participant each placed “Berry Fusion”, “Blueberry”, and “Mango” in the Sweet and Candy category. This category contained eight concept flavors (e.g., “Jazz”, “Silver”, or “Swirl”). Eight participants placed “Jazz” in the Sweet and Candy category; however, one of these participants included the note that they were “not sure what that (Jazz) would be”.

#### 3.2.3. Variation within the Mint Category

There was little variation in the distinct flavor names given in the Mint category, with 77.3% of respondents reporting their favorite cigarillo flavor was “Mint” and 13.6% reporting their favorite cigarillo flavor was “Peppermint”. A single participant each provided the remaining four distinct flavor names in this category: “BLK Smooth”, “Grape”, “Menthol”, and “Mint Fusion”.

#### 3.2.4. Variation within the Alcohol Category

Only 8.9% of participants categorized their favorite cigarillo flavor as “Alcohol”. These participants overwhelmingly preferred “Wine” (65.8%) flavored cigarillos, with four participants specifically noting that they used the “wood tip” variety. For the other 11 flavors in the Alcohol category, two participants each preferred “Red Wine” and “Bourbon”, and one participant each preferred the remaining nine flavors. Although there was a relatively high amount of variation in the flavor names for this category (0.32), many of the flavor names of favorite cigarillos characterized alcoholic beverages, including “Dark Stout”, “White Russian”, and “Spiked Lemonade”. Two of the flavor names in the Alcohol category were concept flavors (“Casino” and “Diamond”), while one included a fruit descriptor (“White Grape”).

#### 3.2.5. Variation within the Menthol Category

While only 5.9% of participants placed their favorite cigarillo flavor in the Menthol category, most favorite responses in that category were “Menthol” (72.0%). Of the other six distinct flavors in this category, two were concept flavors: “Blue” and “Casino.” The Menthol category included three characterizing fruit-descriptor flavors, each selected by one participant: “Berry”, “Blueberry”, and “White Grape”. Two participants placed “Wine” in the Menthol category.

#### 3.2.6. Variation within the Tobacco Category

Of the entire sample, 10.8% of participants placed their favorite cigarillo flavor in the Tobacco category. Most respondents in this category gave a favorite flavor in the “Tobacco/Regular/Plain” group (69.5%). For the 12 remaining flavors placed in the Tobacco category, 9 were concept flavors, including “BLK Smooth”, “Diamond”, “Blue”, “Gold”, “Jazz”, and “Sweet Aromatic.” There were three characterizing flavors outside of the “Tobacco/Regular/Plain” group in this category: “Grape”, “Java Fusion”, and “Wine”.

#### 3.2.7. Variation within the Other Category

The most frequently placed flavor in the Other category was “Diamond” (22.2%), followed by “Tobacco/Regular/Plain” (11.1%) and “Sweet” (11.1%). Ten of the thirteen flavors in the Other category were only selected by one participant. Eight flavors in the Other category were concept flavors, including “Green Leaf”, “Jamaican Blaze”, “Straight Up”, and “Sweet.” The five characterizing flavors in the Other category were “Coffee”, “Honey”, “Russian Cream”, “Tobacco/Regular/Plain”, and “Wine”.

### 3.3. Concept Flavors

Researchers coded 13.1% of the responses as concept flavors (Table 3). The highest percent of concept flavor responses appeared in the Other category (66.7%), followed by the Sweet and Candy (28.2%) and Tobacco (21.7%) categories, whereas most responses in the Mint (97.8%), Fruit (96.0%), and Alcohol (94.7%) categories were characterizing flavors. Out of 66 distinct flavors identified in the sample, 20 (30.1%) were concept flavors. Most flavors in the Tobacco category (69.2%) and the Other category (61.5%) were concept flavors.

When examining the 20 concept flavors, we noted that 8 of the names provided some potential context to their flavor profiles. Three participants placed “Tropical” and “Tropical Fusion” in the Fruit category, and these concept flavor names may indicate a characterizing flavor of tropical fruits. “Arctic Blast” communicates a cooling flavor and was placed in the Sweet and Candy category. Five concept flavors with the descriptor “Sweet” in their name imply a sweet flavor, even if the presence of other additives is unclear. These “Sweet” concept flavors appeared across the Sweet and Candy, Tobacco, and Other categories. The remaining twelve concept flavor names provide less context to predict their flavor profile or whether they were flavored at all, such as “Diamond”, “Blue”, “Casino”, and “Palma”.

### 3.4. Lack of Consensus in Flavor Category Placement

The Fruit category had the smallest percentage of cigarillo flavors that were also placed in other categories (40.0%). All of the Menthol flavor names (100.0%) and most of the Tobacco (76.9%) and Other (69.2%) flavor names also appeared in other categories (Table 4). 

Out of the 66 distinct flavors provided by participants, 20 (29.9%) appeared in more than one category, of which 9 were concept flavors and 11 were characterizing flavors. Overall, nearly twice the proportion of favorite concept flavors appeared in multiple categories (45.0%) compared to favorite characterizing flavors (23.9%) (Table 5).

If participants placed a characterizing cigarillo flavor in multiple categories, most put their responses in the category aligning with that flavor name, as coded by the researchers, with fewer participants placing it in other categories. For example, most “Grape”-preferring participants selected the Fruit category (94.7%), while a single participant each selected the Mint and Tobacco categories. A similar pattern emerged with many of the other repeated characterizing flavors, including “Blueberry” (85.7% of responses in Fruit), “Honey” (94.4% of responses in Sweet and Candy), “Mango” (94.4% of responses in Fruit), “Menthol” (94.7% of responses in Menthol), “Tobacco/Regular/Plain” (91.4% of responses in Tobacco), and “White Grape” (81.8% of responses in Fruit). Participants categorized two characterizing flavors, “Russian Cream” and “Wine”, with less consensus. For example, although 25 of 36 respondents (69.4%) placed “Wine” in the Alcohol category, the other 11 participants placed “Wine” in the Fruit (4), Sweet (2), Menthol (2), and Other (1) categories.

Among concept cigarillo flavors, there was some consensus among participants in placing “Jazz” and “Sweet” (72.7% of responses for both) in the Sweet and Candy category. The remaining seven concept flavors appearing in multiple categories tended to be more evenly distributed. For example, of the eight participants who selected “Diamond” as their favorite flavor, half placed it in the Other category, with the other half selecting the Tobacco (2), Fruit (1), and Alcohol (1) categories. Notably, seven of the nine concept flavors in the Tobacco category also appeared in another flavor category, including “BLK Smooth”, “Blue”, “Jazz”, and “Green Sweet”.

## 4. Discussion

This study found that cigarillo users do not always agree on how to categorize their favorite flavors and that their categorizations may not always align with researchers’ taxonomies. For example, while researchers may agree that wine-flavored cigarillos belong in the Alcohol category [20,21,34], 11% of wine-preferring participants placed it in the Fruit category. Thus, studies comparing Alcohol vs. Fruit cigarillo users could be comparing users of the same flavored product. This finding provides an opportunity to investigate methodological assumptions in measuring and comparing flavor use and preference.

Flavor profiles and additives may vary greatly within categories (e.g., “Spiked Lemonade” vs. “Dark Stout” in the Alcohol category in this study). Tobacco researchers must consider how analytically meaningful it is to group participants based on favorite flavor categories. The current system of classifying the products primarily based on the products’ characterizing flavors, i.e., what they taste and smell like, e.g., fruits, candies, alcoholic drinks, etc., presumes familiarity with or affinity for those items and best explains cigarillo use behavior. With the growing predominance of non-characterizing concept flavors, these categories influence the way researchers sort products, and users must adapt. Future research must further explore which aspects of flavor influence cigarillo initiation and use in order to devise more consistent, salient ways to group users by preference and use.

Concept flavor names not only obscure how a cigarillo is flavored but also whether it is flavored at all. Our study found that 30.0% of participants who categorized their favorite flavor as Tobacco provided either a concept flavor name or a characterizing flavor name that was not tobacco-related. Additionally, 17.4% of responses in the Tobacco category were concept flavors also appearing in other flavor categories. This study did not specifically probe for the brand names of preferred flavors. Different brands may use the same concept flavor names, such “Diamond” by both Game and Swisher Sweets, which may have differences in their taste and smell, leading to variations in how participants categorized these flavors. The non-explicit nature of concept flavor names means multiple products can share the same name with different flavor profiles. Future studies should, therefore, collect both specific flavor and brand information [30] to understand cigarillo users’ flavor preferences and use behaviors.

Cigarillos with names suggesting they are unflavored can also contain flavor additives [35,36]. Some of our participants placed “Tobacco/Regular/Plain” cigarillos in flavor categories, indicating users may perceive cigarillos labeled as “Regular” or “Original” as not unflavored. This observation aligns with prior findings that the level of sweeteners in “Classic” flavored cigarillos can match or exceed some “Sweet” and “Fruit” flavored cigarillos [22]. Sweetened or flavored cigarillos marketed as unflavored may contribute to differences in how users and researchers categorize these products. Based on these findings, it may not be accurate to ask participants whether they regularly use flavored cigarillos to compare behaviors by flavor usage without collecting specific product information.

In the U.S., concept-named cigar products have increased their market share—a trend associated with various policies throughout the country that restrict the sale of flavored cigars [16,32,37,38]. Similar phenomena have appeared in other nicotine products, including electronic vapor products [39,40]. This shift to concept-flavored tobacco products may signal manufacturers’ anticipation of a 2022 U.S. prohibition on the sale and manufacture of cigar products with characterizing flavors, similar to an earlier 2009 U.S. federal law prohibiting characterizing flavors in cigarettes [23,41]. This 2009 legislation did not provide a specific definition of “characterizing”, and manufacturers appear to interpret this term as only a feature of a flavor name rather than discernable experiential aspects of the product, such as taste and smell or the additives used to create them [18,19,38].

Overall, the non-specific nature of concept-named flavors and disagreement among users and researchers on how to categorize flavors confirm the unreliability of studying and regulating flavor based on product names [20,21,22]. To effectively combat the harmful influence of flavored tobacco products, comprehensive flavor regulation policies must focus on the presence of flavor additives [42]. Names can and have been changed to circumvent policy, but restricting additives would prevent companies from continuing to sell flavored cigarillos [43]. 

A potential limitation of our study to consider that it was based on an online survey. Despite implementing quality assurance measures, both in survey design (such as attention check questions [30]), IP address novelty checks, geolocation validation, use of CAPTCHA questions, manual qualitative validation of open-ended screener questions [29], and data cleaning (such as logic checks and minimum response standards [31]), there remains a possibility of misrepresentation by participants of their eligibility. However, due to both the necessary sample size needed to compare categorizations of as many favorite flavor responses as possible and the COVID-19 pandemic, which happened during data collection, in-person data collection methods were not possible.

## 5. Conclusions

This research identifies a lack of consensus within young adult cigarillo users’ categorizations of cigarillo flavors, particularly concept flavored and unflavored products. This finding suggests tobacco researchers must refine how flavor-use data is collected and measured and reconsider why and how flavors are grouped into specific categories. This dissonance in categorization among and between cigarillo users and researchers, as well as the emergence of concept flavors, complicates studying and regulating flavored tobacco use and preference.

## Figures and Tables

**Table 1:** Study sample demographic and tobacco use characteristics (*n* = 426).

Demographic Characteristics ^a^		*n*	%
Gender Identity	Women	235	56.1
	Men	184	43.9
Sexual Identity ^b^	Cisgender Heterosexual Women	88	38.6
	Sexual/Gender Minority Women	140	61.4
	Cisgender Heterosexual Men	153	86.0
	Sexual/Gender Minority Men	25	14.0
Racial Identity	White	160	38.7
	Non-White	253	61.3
Ethnic Identity	Non-Hispanic	329	78.9
	Hispanic	88	21.1
**Tobacco Use History ^c^**		***n***	**%**
Non-Menthol Cigarettes	Ever	310	72.9
	Current	176	41.4
Menthol Cigarettes	Ever	342	80.5
	Current	200	47.6
E-Cigarettes or Vapes	Ever	297	69.7
	Current	196	46.3
Smokeless Tobacco	Ever	114	26.8
	Current	45	10.6
Hookah or Water Pipe	Ever	311	73.0
	Current	124	29.1

^a^ Totals may not add to 426 due to missing responses to demographic questions. ^b^ Sexual and/or gender minority women are oversampled. ^c^ Current tobacco product use is defined as use within the 30 days before taking the survey.

**Table 2:** Preferred cigarillo flavor category placement and variation (*n* = 426).

Flavor Category	Responses % (*n*)	Distinct Flavors in Category *n*	Flavor Variation within Category ^a^
Fruit	41.5% (177)	30	0.17
Sweet and Candy	18.3% (78)	22	0.28
Mint	10.3% (44)	6	0.14
Alcohol	8.9% (38)	12	0.32
Menthol	5.9% (25)	7	0.28
Tobacco	10.8% (46)	13	0.28
Other	4.2% (18)	13	0.72
**Overall**	**100.0% (426)**	**66 ^b^**	**0.15**

^a^ Flavor variation equals Flavors in Category divided by Responses in Category. ^b^ A total of 66 distinct flavors were identified after the removal of repeated flavors in multiple categories.

**Table 3:** Concept flavor responses in flavor categories.

Flavor Category	Concept Flavor Responses % (*n*)	Distinct Concept Flavors *n*	Distinct Concept Flavors/ Total Distinct Flavors %
Fruit	4.0% (7)	6	20.0%
Sweet and Candy	28.2% (22)	8	36.4%
Mint	2.3% (1)	1	16.7%
Alcohol	5.3% (2)	2	16.7%
Menthol	8.0% (2)	2	28.6%
Tobacco	21.7% (10)	9	69.2%
Other	66.7% (12)	8	61.5%
**Overall**	**13.1% (56)**	**20 ^a^**	**30.3%**

^a^ Twenty distinct concept flavors identified after the removal of repeated flavors in multiple categories.

**Table 4:** Percentage of distinct flavors in each category that were also placed in other categories.

Flavor Category	Flavors in Multiple Categories ^a^ *n*	Distinct Flavors *n*	Flavors in Multiple Categories/ Distinct Flavors %
Fruit	12	30	40.0%
Sweet and Candy	10	22	45.5%
Mint	3	6	50.0%
Alcohol	6	12	50.0%
Menthol	7	7	100.0%
Tobacco	10	13	76.9%
Other	9	13	69.2%
**Overall**	**20 ^b^**	**66**	**29.9%**

^a^ Count of distinct flavors within flavor categories that were also placed in other categories. ^b^ A total of 20 distinct flavors in multiple categories after the removal of repeats.

**Table 5:** Individual cigarillo flavors placed in more than one flavor category.

Flavors in Multiple Categories	Responses per Category						
	Fruit	Sweet	Mint	Alcohol	Menthol	Tobacco	Other
Berry	5				1		
BLK Smooth *	1		1			1	
Blue *					1	1	
Blueberry	12	1			1		
Casino *				1	1		
Diamond *	1			1		2	4
Grape	36		1			1	
Green Sweet *		1				1	
Honey		17					1
Irish Cream	1			1			
Jazz *	1	8				1	1
Mango	17	1					
Menthol			1		18		
Tobacco/Regular/Plain		1				32	2
Russian Cream	1	3		1			1
Silver *	1	1					1
Sweet *		8				1	2
Sweet Aromatic *						1	1
White Grape	9			1	1		
Wine	4	2		25	2	2	1

* Indicates concept flavor.

## Data Availability

The data presented in this study are available on request from the corresponding author. The data are not publicly available due to ongoing analyses by the study team.

## Supplementary Materials

The following supporting information can be downloaded at: https://www.mdpi.com/article/10.3390/ijerph19127219/s1, Table S1: Valid Preferred Cigarillo Flavors by Participant-Selected Categories.
