# Supplementary material for: Dissonance in Young Adult Cigarillo Users’ Categorization of Concept Flavored and Unflavored Products
Source: Int J Environ Res Public Health. 2022 Jun 13;19(12):7219. doi: 10.3390/ijerph19127219 (PMC9223595; doi:10.3390/ijerph19127219)
Supplement: Supplementary file 1 [file ijerph-19-07219-s001.zip › ijerph-1744602-supplementary.pdf]

Table S1: Valid Preferred Cigarillo Flavors by Participant-Selected Categories

Fruit	Sweet	Mint	Alcohol	Menthol	Tobacco	Other
Apple (4)	**Arctic Blast (1)**	**Blk Smooth (1)**	Black Russian (1)	Berry (1)	**Blk Smooth (1)**	Coffee (1)
Banana Smash (2)	Berry Fusion (1)	Grape (1)	Bourbon (2)	**Blue (1)**	**Blue (1)**	**Diamond (4)**
Berry (5)	**Black Sweet (1)**	Menthol (1)	**Casino (1)**	Blueberry (1)	**Diamond (2)**	**Green Leaf (1)**
Black Cherry (1)	Blueberry (1)	Mint (34)	Dark Stout (1)	**Casino (1)**	**Gold (1)**	Honey (1)
**Blk Smooth (1)**	Candy (3)	Mint Fusion (1)	**Diamond (1)**	Menthol (18)	Grape (1)	**Jamaican Blaze (1)**
Blue Raspberry (5)	Chocolate (2)	Peppermint (6)	Irish Cream (1)	White Grape (1)	**Green Sweet (1)**	**Jazz (1)**
Blueberry (12)	Clove (1)		Red Wine (2)	Wine (2)	Java Fusion (1)	Regular (2)
Cherry (17)	French Vanilla (1)		Russian Cream (1)		**Jazz (1)**	Russian Cream (1)
**Diamond (1)**	**Green Sweets (1)**		Spiked Lemonade (1)		**Red Sweet (1)**	**Silver (1)**
Grape (36)	Honey (17)		White Grape (1)		**Sweet (1)**	**Straight up (1)**
Irish Cream (1)	Honey Bourbon (1)		White Russian (1)		**Sweet Aromatic (1)**	**Sweet (2)**
**Jazz (1)**	**Jazz (8)**		Wine (25)		Wine (2)	**Sweet Aromatic (1)**
Mango (17)	Mango (1)					Wine (1)
Orange (1)	**Palma (1)**				No Flavor (6) / Non flavored (2) / Original (2) / Plain (4) / Regular (5) / Tobacco (7) / Unflavored (2) = Total (32)	
Peach (6)	Plain (1)					
Pineapple (7)	Russian Cream (3)					
Raspberry (1)	**Silver (1)**					
Red Berry (4)	**Sweet (8)**					
Russian Cream (1)	**Swirl (2)**					
**Silver (1)**	Vanilla (20)					
Strawberry (29)	White Cream (1)					
Strawberry Harvest (1)	Wine (2)					
Sweet Cherry (1)						
**Tropical (2)**						
**Tropical Fusion (1)**						
Watermelon (2)						
White Grape (9)						
White Peach (3)						
Wild Berry (1)						
Wine (4)						

Bold indicates concept flavor; participants (*n* = 426) provided and named 66 unique flavors as their preferred flavors, placed across seven flavor categories. The number next to each flavor name indicates the number of participants who placed that flavor in that specific category.
